# Diurnal variation of NMR based blood metabolites in calves fed a high plane of milk replacer: a pilot study

**DOI:** 10.1186/s12917-017-1185-2

**Published:** 2017-08-23

**Authors:** Morteza H. Ghaffari, Jayden A. R. MacPherson, Harma Berends, Michael A. Steele

**Affiliations:** 1 Department of Agricultural, Food, and Nutritional Science, University of Alberta, Edmonton, T6G 2P5 Canada; 2 Trouw Nutrition R&D, P.O. Box 220, 5830 AE Boxmeer, The Netherlands

**Keywords:** Calf, Plasma, Metabolomics, NMR spectroscopy

## Abstract

**Background:**

Blood profiles have been used to monitor herd health status, diagnose disorders, and predict the risk of diseases in cattle and calves. Characterizing plasma metabolites in dairy calves could provide further insight into daily metabolic variations and the mechanisms that lead to metabolic diseases. In addition, by understanding physiological ranges of plasma metabolites relative to meal and the time of feeding in healthy animals, veterinarians can accurately diagnose abnormalities with a blood test. For diagnostic purposes, nuclear magnetic resonance (NMR) spectroscopy shows promise as a new and reliable method to determine a large number of blood metabolites simultaneously.

**Results:**

Results demonstrated that the concentration of specific metabolites in plasma (i.e., lysine, isoleucine, leucine, tyrosine, glutamine, creatine, and 1-methylhistidine) fluctuated around meal times, while others (i.e., glutamic acid, methanol, formic acid, and acetic acid) maintained a stable temporal concentration. In addition to temporal changes in concentration, results also characterized differences for overall plasma metabolite concentrations; for example, methionine had the lowest (38 μM) while glutamine had the highest concentration (239 μM) amongst plasma AA. This is the first report describing how the plasma metabolome changes during 24-h period in young calves fed an elevated plane of milk replacer twice daily.

**Conclusions:**

Data from this pilot study will help to establish reference standards for future metabolic diagnostics in dairy calves. In addition, this pilot study illustrated that feeding milk replacer may influence plasma metabolite concentrations. With the rapid implementation of blood metabolomics in monitoring animal health, it is then important to consider the time of feeding during the day when interpreting metabolomics analysis results.

**Electronic supplementary material:**

The online version of this article (doi:10.1186/s12917-017-1185-2) contains supplementary material, which is available to authorized users.

## Background

Plasma metabolites are widely used to monitor the health and metabolic status of cattle herds [1]. Nuclear magnetic resonance (NMR) spectroscopy is a method that can be applied to identify and quantify many metabolites in biological samples, including blood. The minimal sample preparation and high analytical reproducibility along with the non-destructive characteristics of NMR spectroscopy represents significant advantages for employing NMR in the field of metabolomics research compared with other methods [2]. Identification of metabolomic profiles using NMR that relate to specific outcomes such as metabolic disorders and diseases could improve the speed of diagnosis and treatments [3]. Reported metabolite research values vary in sampling times during the day as well as the physiological stage of the animal. This represents a serious knowledge gap for veterinary diagnostics as the plasma concentrations of some metabolites, such as non-esterified fatty acids, glucose, and amino acids (AA), have been shown to be affected by time of feeding in cattle [4, 5]. This is important to consider for clinical investigations to avoid misinterpretation of results due to the meal effects.

Blood metabolite concentrations may also fluctuate throughout the day, therefore sampling time differences between studies may be a primary reason for the discrepancies in published literature. Having temporal reference data for metabolite concentrations of dairy calves could further strengthen our understanding of physiologically healthy concentrations versus metabolic disorders (i. e., chronic diarrhea, pneumonia, and insulin sensitivity). Moreover, most of the currently available data describing metabolic responses comes from studies in adult cattle, and these findings may not represent metabolomic profiles of calves. Therefore, it is important to understand and characterize the metabolome of calves to establish physiologically healthy fluctuations in metabolites throughout the day.

In dairy calves, it has been shown that increasing meal size results in slower gastric emptying [6]. The rate at which nutrients empty from the abomasum can also influence the rate at which those nutrients are delivered to the small intestine and absorbed into the bloodstream [7]. Therefore, the objective of this report is to describe diurnal variations of plasma metabolites in dairy calves fed an elevated plane of milk replacer twice daily using NMR spectroscopy. In this pilot study, NMR-based metabolomic analysis technology was used because it allowed us to study a large number of metabolites in the blood of dairy calves. To the best of our knowledge, this is the first time that a 24-h period of plasma metabolome has been characterized by NMR spectroscopy in calves fed an elevated plane of nutrition.

## Results

All calves were clinically healthy at time of sampling. The mean daily starter feed intake and milk replacer intake from day 19 to day 23 of age were 46.0 ± 21.4 g/day and 7.51 ± 0.49 L/day, respectively.

### Nuclear magnetic resonance spectroscopy

**Fig. 1:**
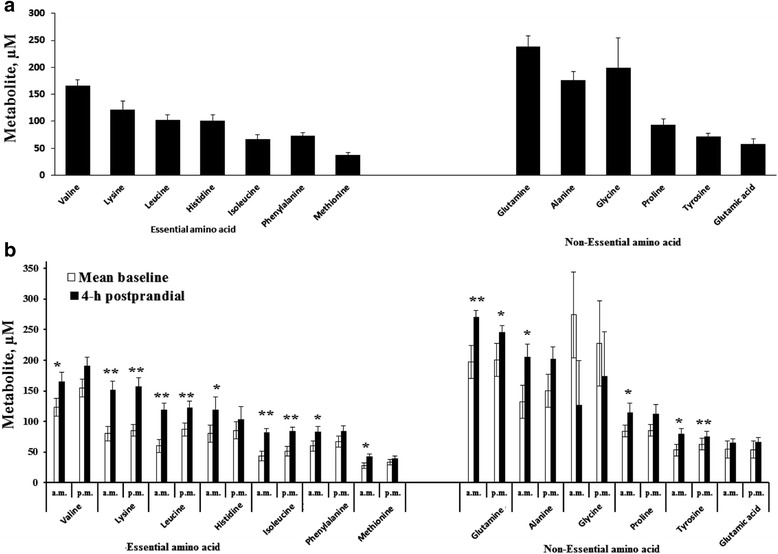
Twenty-four hour mean values (**a**), mean baseline and 4-h postprandial concentrations (**b**) of blood essential and non-essential amino acids in Holstein calves fed elevated plane of nutrition twice daily. Data are expressed as means ± SE. For each time point, * = significant difference at *P* < 0.05; ** = significant difference at *P* < 0.01

Twenty-four hour mean values of the plasma essential amino acids (EAA; histidine, lysine, methionine, phenylalanine, valine, leucine, and isoleucine) and nonessential amino acids (NEAA; alanine, glutamine, glutamic acid, glycine, proline, and tyrosine) are presented in Fig. 1a. Glutamine had the highest concentration (238.6 μM) while methionine was the lowest (38 μM) amongst plasma AA. Valine and lysine were the most abundant essential plasma AA. Mean baseline and four-hour postprandial plasma concentrations of AA are presented in Fig. 1b. A four-hour postprandial sampling time was selected based on the knowledge that the soluble components of milk enter the small intestine within 2 to 3 h after a meal [8]. In the morning meal, the 4-h postprandial plasma concentrations of histidine, lysine, leucine, isoleucine, valine, methionine, phenylalanine, alanine, proline, tyrosine, and glutamine were significantly higher (*P* < 0.05) compared with baseline. In the evening meal, the 4-h postprandial plasma concentrations of isoleucine, lysine, tyrosine, and glutamine were significantly higher (*P* < 0.05), and valine (*P* = 0.07) and alanine (*P* = 0.08) tended to be higher compared with baseline. The plasma concentrations of glutamic acid and glycine did not significantly change in response to time of meal feeding. The patterns of diurnal variation of plasma EAA and NEAA are displayed in supplementary file (Additional files 1 and 2).

**Fig. 2:**
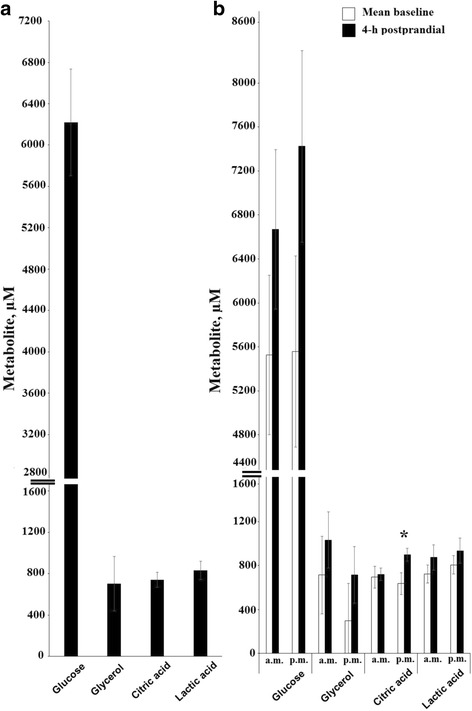
Twenty-four hour mean values (**a**), mean baseline and 4-h postprandial concentrations (**b**) of blood glucose, glycerol, citric acid, and lactic acid in Holstein calves fed elevated plane of nutrition twice daily. Data are expressed as means ± SE. For each time point, * = significant difference at *P* < 0.05

**Fig. 3:**
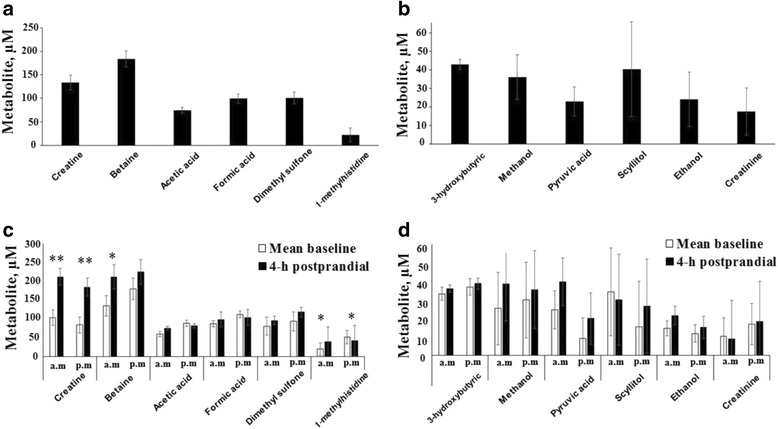
Twenty-four hour mean values (**a, b**) and mean baseline and 4-h postprandial concentrations (**c, d**) of blood metabolites in Holstein calves fed elevated plane of nutrition twice daily. Data are expressed as means ± SE. For each time point, * = significant difference at *P* < 0.05; ** = significant difference at *P* < 0.01

The plasma AA concentrations varied markedly according to the time of day. Twenty-four-hour mean plasma glucose, glycerol, citric acid, and lactic acid concentrations are shown in Fig. 2a. Mean baseline and 4-h postprandial plasma recordings of glucose, glycerol, citric acid, and lactic acid concentrations are shown in Fig. 2b. Twenty-four hour mean concentrations of other plasma metabolites such as creatine, betaine, acetic acid, formic acid, dimethyl sulfone, 1-methylhistidine, 3-hydroxybutyric acid, methanol, pyruvic acid, scyllitol, ethanol, and creatinine are presented in Fig. 3a and b. Mean baseline and 4-h postprandial plasma recordings of other metabolite concentrations are presented in Fig. 3c and d.

The 4-h postprandial plasma concentrations of betaine (in the morning meal) and citric acid (in the evening meal) were significantly higher (*P* < 0.05) compared to their baseline. Plasma concentration of glucose tended to be higher (*P* = 0.10) at 4-h postprandial compared to baseline after both meal feedings. Comparison of postprandial plasma concentrations of 1-methylhistidine (*P* < 0.05) and creatine (*P* < 0.01) after both meal feedings revealed significantly higher concentrations at 4-h postprandial compared to their baseline. The patterns of diurnal variation of other plasma metabolites are displayed in an additional file (Additional file 3). Plasma concentration of creatine significantly increased after feeding to reach a maximum level of 222.3 μM in less than 3 h, followed by a progressive decrease until the next feeding. After the morning feeding, the plasma glycerol concentration numerically increased and there were small fluctuations throughout the day. Plasma concentration of pyruvic acid numerically increased after the morning meal, but its concentration significantly decreased after the evening meal. The plasma concentrations of other metabolites such as acetic acid, formic acid, 1-methylhistidine, dimethyl sulfone, 3-hydroxybutyrate, methanol, scyllitol, ethanol, and creatinine did not significantly change in response to time of meal feeding.

## Discussion

Metabolomics is becoming an important tool in clinical research, allowing for very early and accurate diagnosis of disorders and diseases [9]; however, this technique is not implemented on most of dairy farms as a tool to selectively tailor management decisions in calves and cattle. The plasma metabolite profiles in dairy calves have not been properly characterized. Thus, this pilot study specifically focused on measuring the postprandial and diurnal variation of blood metabolites using NMR in three male Holstein dairy calves fed an elevated plane of milk replacer twice daily.

### Amino acids

For dairy calves, milk provides most of the nutrients, such as the AA, needed to support adequate growth for young calves before they are able to digest solid feeds [10]. The postprandial concentrations of all EAA and some NEAA in the plasma significantly increased after ingestion of milk replacer in this pilot study. The observed difference in the plasma level of EAA after milk feeding is in agreement with previously reported data, indicating that the plasma EAA concentrations increased following milk feeding in pre-weaned calves [11]. In this study, the different postprandial concentrations of plasma AA and the degree of increase after the meal can partially be explained by different AA digestibility [12]. Plasma glutamine levels increased after MR feeding in this pilot study, which is consistent with the results of McCormick and Webb [13] who reported that glutamine was the only amino acid increased after feeding in plasma of calves. In contrast, Hammon and Blum [10] reported no change in the plasma glutamine concentration in calves fed only milk replacer during the first week of life. Glutamine was the most abundant free AA in calf plasma, which is consistent with previous findings underlining the important role of glutamine in maintaining net protein synthesis and accretion [14]. Studies on dietary glutamine utilization in human indicated at least 50% of the dietary glutamate and glutamine is taken up by the GIT and is completely oxidized to CO_2_ [15]. This means that the plasma glutamine concentration of calves in this study may be affected by the utilization and metabolism of glutamine by small-intestine.

In contrast to all other post-prandial results, the plasma concentration of glycine was numerically lower than the baseline level 4 h after the meal. Glycine is synthesized in the body through various complex pathways involving serine, threonine, choline, and hydroxyproline via inter-organ metabolism involving primarily the liver and kidneys [16]. The lower postprandial glycine concentration in plasma of dairy calves might be associated with low concentration of glycine in milk [17, 18]. Wang et al. [16] reported that the intake of glycine from milk provides at most only 20% of the glycine required by the 7-day old piglet; thus, 80% of the glycine needed by the piglets must be provided through endogenous synthesis.

The plasma AA concentrations in dairy calves are likely more affected by diets than mature cows. Halfpenny et al. [19] reported that plasma AA concentrations in dairy cows are relatively constant during the day. In addition, Huntington [20] showed that AA absorption in lactating cows was relatively constant until 12 h after feeding, likely because of the continuous outflow of microbial and dietary protein from the rumen, which is a key difference between pre-weaned calves and more mature weaned animals. Therefore, the results of this study indicated that the plasma AA status of young calves is highly related to milk replacer intake.

### Additional metabolites

Glucose, lactic acid, citric acid, and glycerol were the most abundant metabolites in the plasma of the dairy calves in this report. The concentration of glucose in the calves in this study were within the range suggested for plasma glucose concentrations in dairy calves fed high plane of milk replacer at 4 week of age, which vary between 6500 and 8000 μM/L [6]. The postprandial concentrations of glucose in the plasma tended to increase after ingestion of milk replacer in this pilot study, which is in line with the results of previous studies [6, 21]. Plasma glucose concentration can be affected by the level of milk feeding, milk carbohydrate sources [22], endogenous glucose production from gluconeogenesis in the liver [23], and age [24]. As calves age, the concentration of plasma glucose decreases, which is mainly due to a shift in the primary source of energy from glucose to volatile fatty acid [25]. Therefore, milk feeding can be considered an important factor for influencing the postprandial plasma glucose concentration in dairy calves and should be considered when evaluating glucose concentrations in calves.

Lactic acid was the most abundant organic acid in the plasma of these calves. A post-prandial rise of lactate concentrations in the plasma has been previously reported in milk-fed calves [26, 27], although in this pilot study the rise was not significant in the calves. The accumulation of lactic acid is an important contributory factor to metabolic acidosis in neonatal calves with diarrhea in the previous study [28]. So far, limited information is available regarding the plasma concentration of citric acid in dairy calves. Citric acid is formed from acetyl-CoA and oxaloacetic acid in a reaction catalyzed by citrate synthase [29]. In this study, the plasma citric acid level increased after the evening meal. Reasons for the increased plasma level of citric acid following evening milk feeding in calves are unclear.

In this pilot study, plasma concentrations of creatine, betaine, and 1-methylhistidine were affected by time of meal feeding in calves. The plasma creatine concentrations were found to increase after each meal. Creatine is a nitrogenous amine found in muscle tissue that is involved in ATP synthesis and muscle function [30]. The elevation of the plasma creatine concentrations following the consumption of a milk replacer meal can be mainly due to the breakdown of proteins supplied by the milk replacer. Based on NMR spectroscopy, Basoglu et al. [3] reported that the concentration of plasma creatine increased in neonatal calves with diarrhea compared with healthy calves because of enhanced intestinal barrier function. The postprandial concentration of betaine in the plasma was also found to increase after the morning meal in this pilot study. Plasma betaine concentration in human beings can be affected by absorption from the intestine, synthesis from choline, metabolism to dimethylglycine, storage in muscle, liver and kidney tissues, as well as urinary elimination [31]. However, the factors causing the postprandial level of betaine rise in plasma are unclear. Plasma concentration of 1-methylhistidine observed in dairy calves also increased and was four times higher than those in a mature cow [32]. However, in this study, the postprandial increase of 1-methylhistidine may have been a consequence of increased breakdown of milk proteins. Therefore, it can be concluded that the diurnal variations of some plasma metabolites (i.e., acetic acid, formic acid, ethanol, and creatinine) were independent of meal feeding whereas the diurnal variations in most AA, creatine, 1-methylhistidine in plasma were strongly affected by meal feeding in calves.

### Limitations

This study was a preliminary investigation of the diurnal variation of blood metabolites in 20 day old calves fed milk replacer. The major limitation of this pilot study was biological replication, and the findings could be very specific to this age group and this feeding regimen. However, this pilot study will provide the groundwork for further studies investigating post-prandial responses of blood metabolites in calf feeding programs.

## Conclusion

Metabolomic profiling is an emerging diagnostic tool based on the comprehensive determination of many metabolites contained in bio-fluids and in biological samples. This pilot study indicated that some plasma metabolites exhibit a definite pattern and change with time relative to milk replacer feeding. These findings will be useful in guiding the design and interpretation of future metabolite-based studies in calves. Future metabolomics studies with larger sample sizes and different ages are required to verify the findings of this pilot study, and to explore its clinical application for the diagnosis and treatment of diseased animals.

## Methods

This pilot study was conducted at the Trouw Nutrition Ruminant Research Centre (Boxmeer, The Netherlands). All procedures and sampling were approved by the Animal Care and Use Committee of Utrecht University (2014.III.04.045). Three Holstein bull calves (average age 20.0 ± 1.0 days) weighing 56.0 ± 3.3 kg were housed in individual hutches (1.07 × 1.60 m) bedded with wheat straw. All calves received reheated dam colostrum from a bottle (4 L divided into two feedings at 1 and 6 h of birth). All calves were fed milk replacer (150 g/L; 24% crude protein (skimmed milk powder), 18% crude fat, and 45% lactose; %1.83 lysine, %0.55 methionine, and %0.83 cystine + methionine, Sloten B.V. Deventer, The Netherlands) from nipple buckets. Calves were stepped up to 8 L/day of milk replacer during the first week of life which consisted of a total of 2.5 L for day 2 and 3, 3.0 L for day 4 and 5, and 3.5 L for day 6 and 7. From day 8 onward calves were fed a high plane of nutrition of 8 L/day which was divided equally and offered in two daily feedings (07:00 h and 17:00 h). Calves had ad libitum access to water, pelleted calf starter (18.2% crude protein, 11.2% crude fibre, 2.2% crude fat, 3 mm pellet; AgruniekRijnvallei, Wageningen, The Netherlands), and wheat straw (3 cm chop length), provided in buckets in the front of the hutch. Jugular catheters (Intraflon 2 13G, Ecouen, France) were inserted in the morning (08:00 am) of the sampling day. Starting at 12:00 pm, blood samples were collected every hour for 24 h via the jugular catheters in EDTA (10 ml) vacutainers. Immediately after each collection, all catheters were flushed with a 2% heparinized saline solution. Blood samples were immediately centrifuged at 2772×*g* for 30 min, after which the plasma was separated, frozen at −20 °C and shipped on dry ice to The Metabolomics Innovation Centre at the University of Alberta, Canada for NMR spectrum analysis. The NMR samples were prepared as described by Psychogios et al. [33]. All ^1^H–NMR spectra of blood samples were collected on a 500 MHz (Varian Inc., Palo Alto, CA, USA) spectrometer equipped with either a Z-gradient PFG Varian cold-probe or a 5 mm HCN Z-gradient pulsed-field gradient (PFG) room-temperature probe. The resulting ^1^H–NMR spectra were processed and analyzed using the Chenomx NMR Suite Professional software package version 6.0 (Chenomx Inc., Edmonton, AB, Canada), as previously described [34].

### Statistical analysis

Data of the plasma metabolome are presented as means ± standard error of the mean, obtained using PROC MEANS of SAS [35]. T-tests were used to determine the significance of changes between baseline and four-hour postprandial. The mean baseline is the average of the metabolite concentration 2 h before the meal (at 7:00 h and 17:00 h).

## Additional files

Additional file 1: Concentrations of blood essential amino acids measured at hourly interval for 24 h from 05:00 to 04:00 h. Feeding times are marked with an arrow. Data are expressed as means ± SE. (DOC 426 kb)

Additional file 2: Concentrations of blood non-essential amino acids measured at hourly interval for 24 h from 05:00 to 04:00 h. Feeding times are marked with an arrow. Data are expressed as means ± SE. (DOC 189 kb)

Additional file 3: Concentrations of blood metabolites with response to meal feeding times of 7:00 and 17:00 measured at hourly interval for 24 h. Feeding times are marked with an arrow. Data are expressed as means ± SE. (DOC 724 kb)

## Abbreviations

AA: Amino acids
BW: Body weight
EAA: Essential amino acids
GIT: Gastrointestinal tract
NEAA: Nonessential amino acids
NMR: Nuclear magnetic resonance
TCA: Tricarboxylic acid
