# Supplementary figures and images for: Diurnal variation of NMR based blood metabolites in calves fed a high plane of milk replacer: a pilot study
Source: BMC Vet Res. 2017 Aug 23;13:271. doi: 10.1186/s12917-017-1185-2 (PMC5569568; doi:10.1186/s12917-017-1185-2)

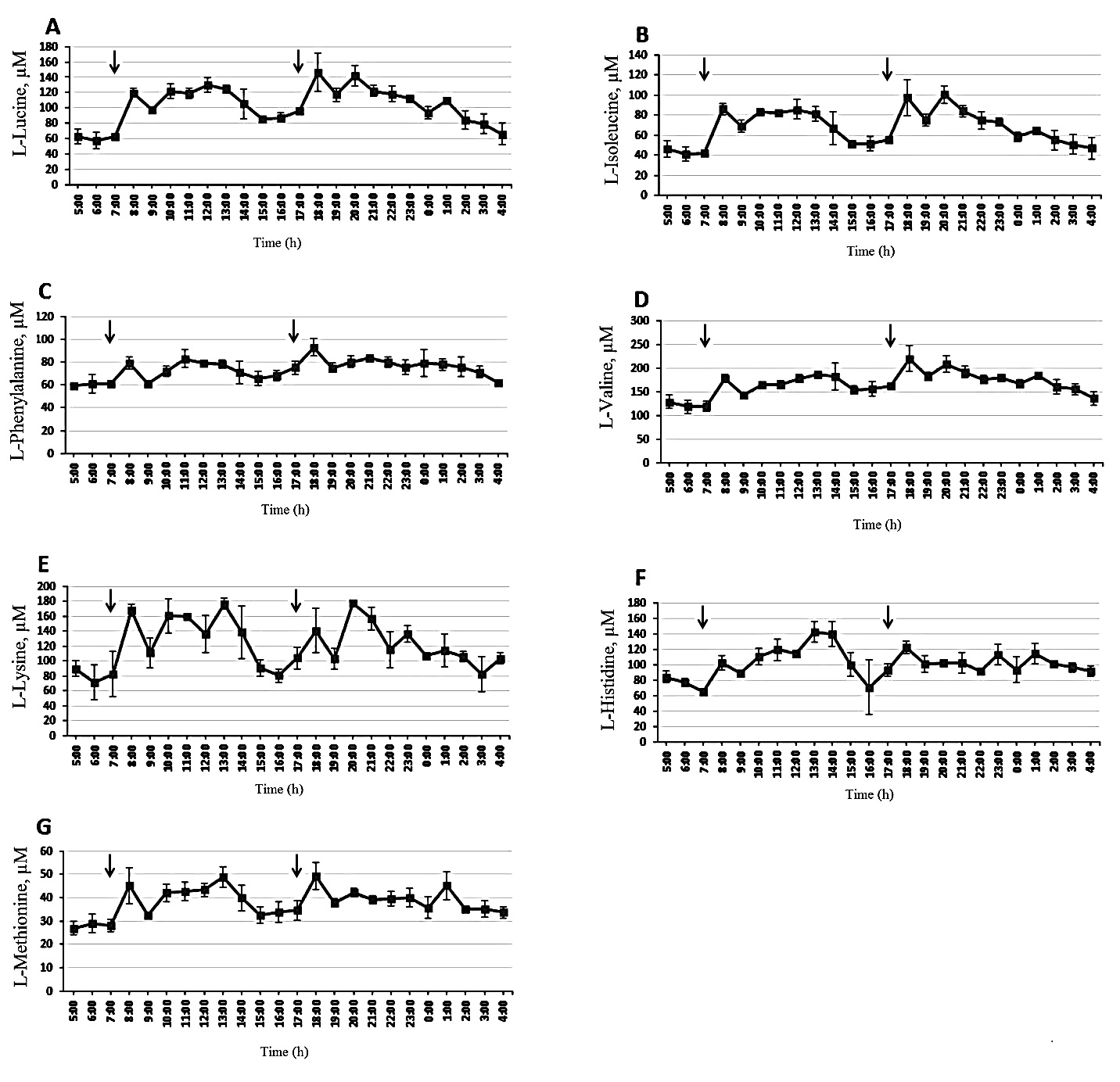

Supplement: Supplementary file 1 — Concentrations of blood essential amino acids measured at hourly interval for 24 h from 05:00 to 04:00 h. Feeding times are marked with an arrow. Data are expressed as means ± SE. (DOC 426 kb) [file 12917_2017_1185_MOESM1_ESM.doc]

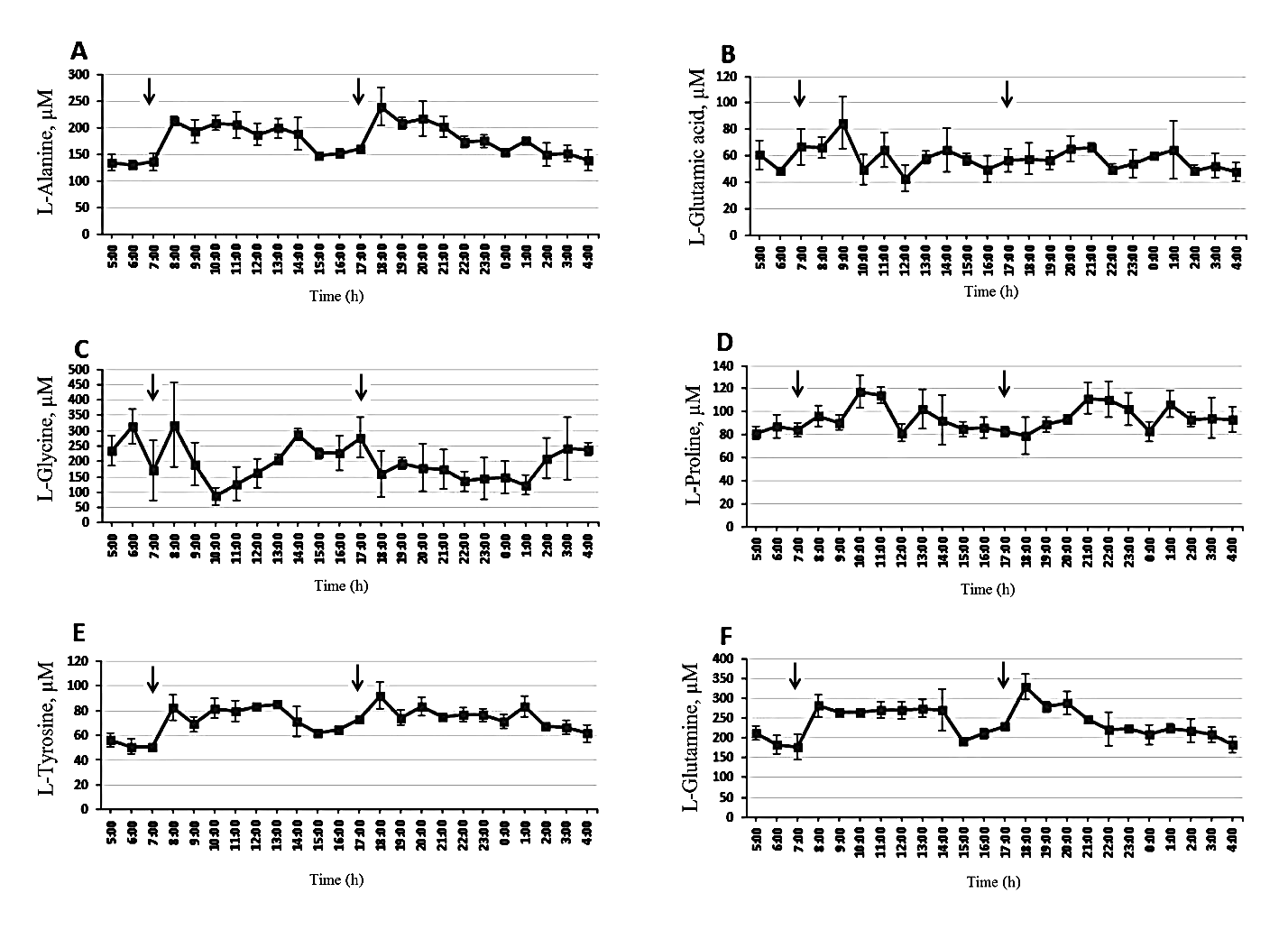

Supplement: Supplementary file 2 — Concentrations of blood non-essential amino acids measured at hourly interval for 24 h from 05:00 to 04:00 h. Feeding times are marked with an arrow. Data are expressed as means ± SE. (DOC 189 kb) [file 12917_2017_1185_MOESM2_ESM.doc]

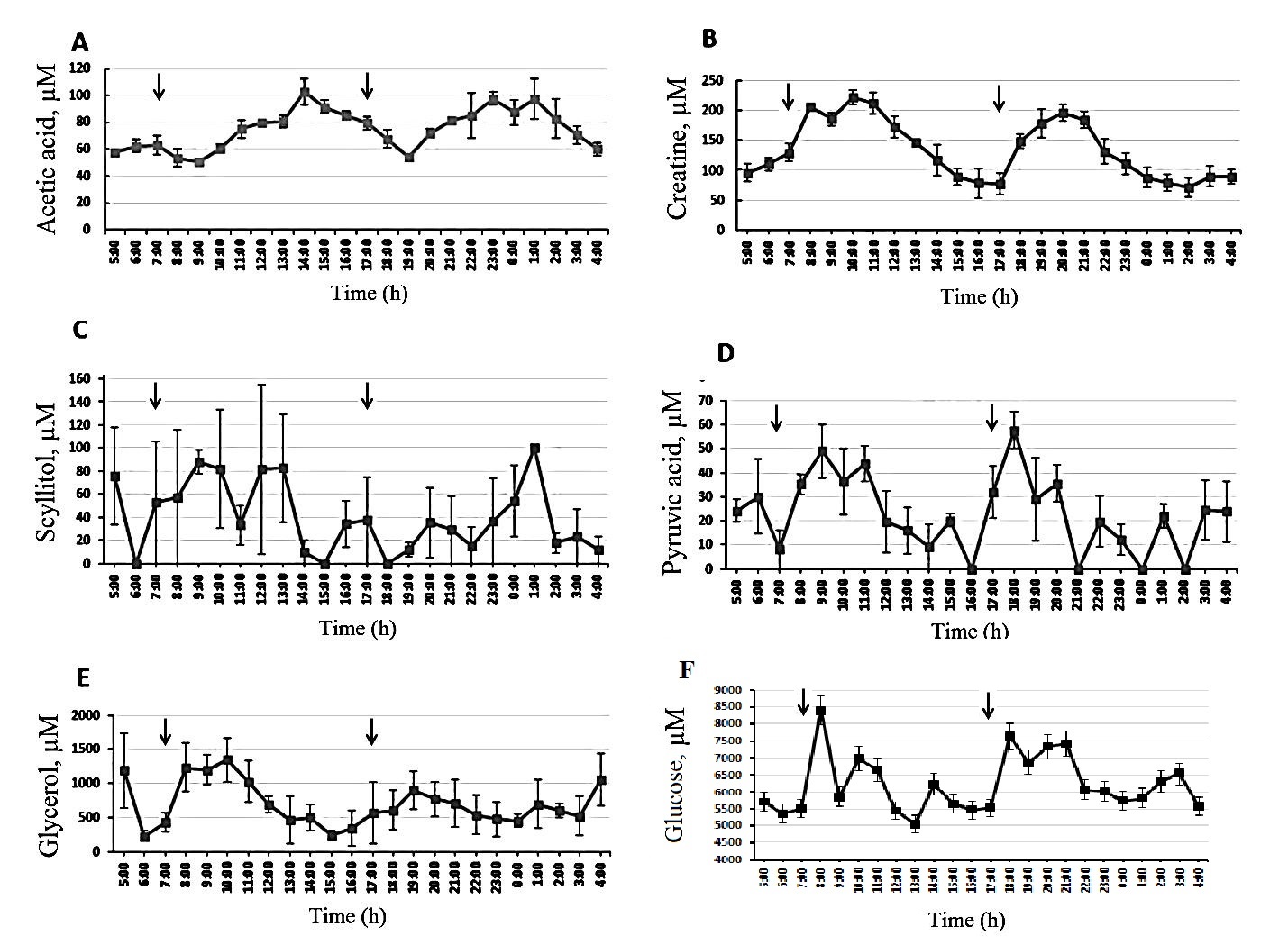


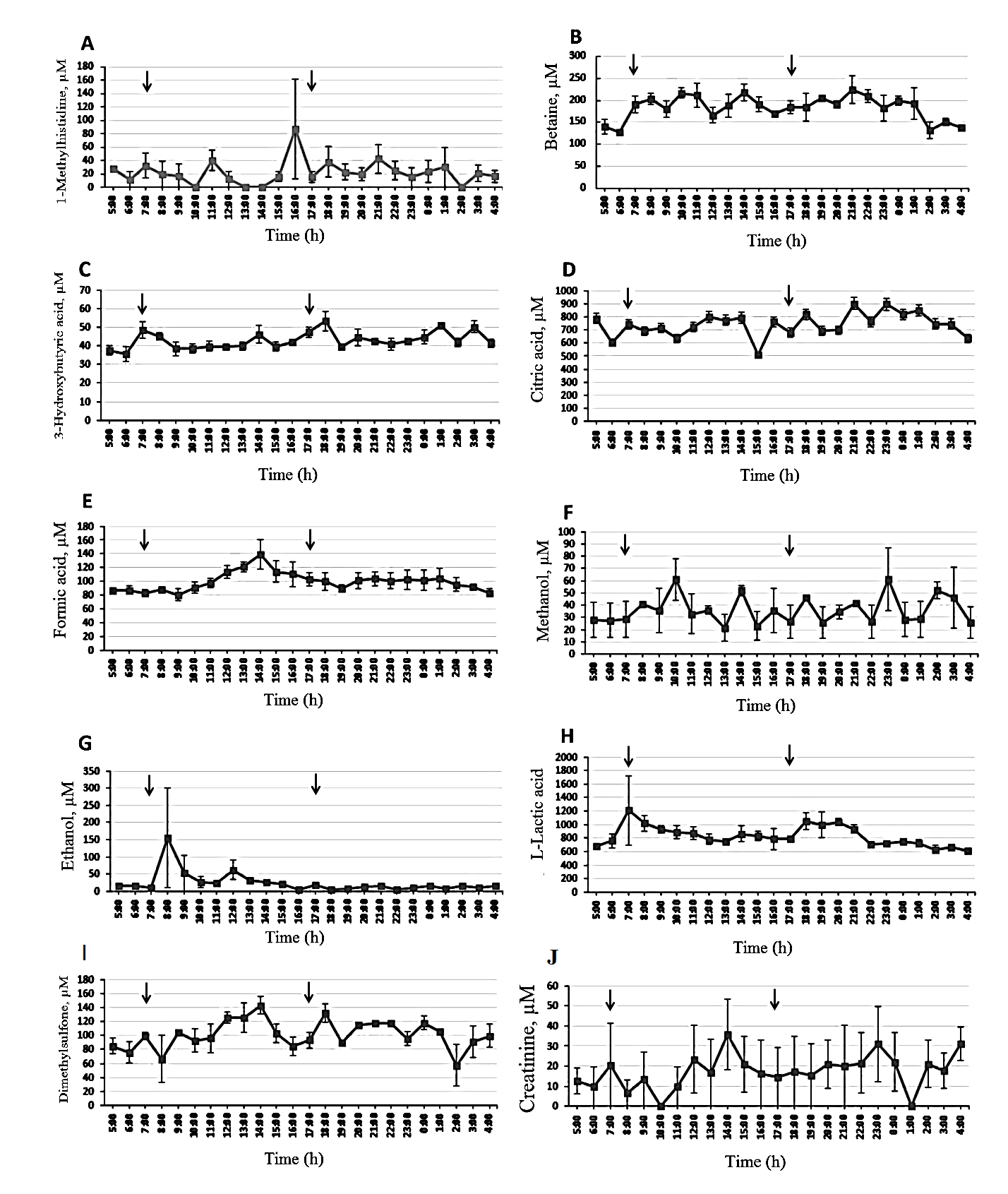

Supplement: Supplementary file 3 — Concentrations of blood metabolites with response to meal feeding times of 7:00 and 17:00 measured at hourly interval for 24 h. Feeding times are marked with an arrow. Data are expressed as means ± SE. (DOC 724 kb) [file 12917_2017_1185_MOESM3_ESM.doc]
